# Studying the Pyroelectric Effects of LiNbO_3_ Modified Composites

**DOI:** 10.1186/s11671-020-03341-w

**Published:** 2020-05-12

**Authors:** Fan Zhang, Hua Kang, Yixuan Lin, Li Guan, Hüsnü Aslan, Meining Zhang, Lin Niu, Mingdong Dong

**Affiliations:** 1 Department of Chemistry, Renmin University of China, Beijing, 100872 People’s Republic of China; 2 Sino-Danish Center for Education and Research (SDC), interdisciplinary Nanoscience Center (iNANO), Aarhus University, Dk-8000 Aarhus C, Denmark; 3 School of Materials Science & Engineering, Nanyang Technological University, Singapore, 639798 Singapore

**Keywords:** Pyroelectric effects, Self-assembly of nanoparticles, Composite film

## Abstract

LiNbO_3_ (LN) crystal has been widely used as a pyroelectric material due to its spontaneous electric polarization, which could be recharged easily and can directly convert heat energy into electricity. LN crystal’s heat-resistant, low-cost, and low dielectric loss properties make it possible for its applications in room-temperature pyroelectric devices and thermal sensors. However, LN crystal suffers from fragility, inflexibility, and other mechanical properties, which limit its suitability for many applications in various fields. In this study, the LN modified flexible pyroelectric films, composed of LN micro-particles, polypropylene (PP) matrix, and multiwalled carbon nanotubes (MWCNTs), are successfully fabricated. The pyroelectric effects of LN crystal and LN/PP/MWCNT composite films are characterized by monitoring the patterned self-assembly of nanoparticles and the output pyroelectric currents. The excellent pyroelectric properties of the composites have potential applications in energy harvesters or sensors.

## Introduction

Research on the pyroelectric effect has been greatly promoted with the rapid development of new technologies such as laser and infrared scanning imaging [1–4]. The investigation on the pyroelectric effect and related phenomena in various ferroelectric materials (FEM) are used for the generation of pyroelectric convertors for various purposes including single and multi-element pyroelectric detectors of radiation (PDR) [5–7]. Many pyroelectric detectors and camera tubes with excellent performance have been developed [8–10]. In addition, it has also been reported that the pyroelectric effects are used to collect heat in the environment [11–14], rotation rate sensing [15], and gas sensing substrate [16, 17].

As a kind of ferroelectric material, LiNbO_3_ (LN) has attracted great attention due to its large nonlinear optical coefficient to be used as nonlinear optical materials with a high Curie temperature (T_c_, ~ 1413 K) and melting point (T_m_, ~ 1523 K) [18–20]. The polar crystal structure of LN crystals exhibits spontaneous polarization that can be changed by temperature variations [21, 22]. And the nonlinear optical coefficients were linear functions of spontaneous polarization, which are temperature dependence of polarization and are of prime importance in nonlinear research [23]. The spontaneous electric polarization properties of FEM enable it recharge with ease and can directly convert thermal energy into electricity [24].

Among the reported pyroelectric materials such as PZT and Polyvinylidene fluoride (PVDF), barium titanate (BaTiO_3_) [25–27], lead-based materials are the most widely used traditional pyroelectric materials. However, the reported toxicity, high costs and possible polltion to environment limit their application in many fields. Therefore, high-performance and lead-free pyroelectric materials have attracted widely attentions [28]. As a kind of lead-free ferroelectric crystal, LN shows a high pyroelectric coefficient, low dielectric loss [29], which make it feasible to be used as pyroelectric devices with higher sensitivity, and good stability. However, the fragility, inflexibility, and the difficulty in reprocessing of the LN bulky crystal wafer limit its application in many fields [30]. Therefore, improvement of its mechanical properties is of critical importance.

Herein, we report the fabrication and characterization of polymer-based composites, which incorporate the pyroelectric properties of LN crystal and mechanical advantages of the polymer simultaneously. The LN particles modified flexible pyroelectric composite film based on polypropylene (PP) matrix is fabricated, in which the LN microparticles and the multiwalled carbon nanotubes (MWCNTs) are adopted as fillers. The PP polymer has many advantages such as low-cost, flexibility, and low dielectric loss, which is suitable to be used as the matrix of the composite [31]. Moreover, as a typical thermoplastic polymer, PP matrix could be processed into thin film by hot-pressing. LN particles are the key components since they exhibit excellent pyroelectric effect when the particle sizes are restricted in certain range [32, 33]. The MWCNTs are adopted as the conductive elements to improve the electrical profile of the composite matrix. Therefore, the composite has incorporated excellent mechanical properties of PP matrix and the superior pyroelectric effects of LN nanoparticles [34–36].

## Methods

### Materials

All materials and chemicals were purchased commercially and used as received. LN wafer was fabricated and purchased from the Shanghai Institute of Optics and Fine Mechanics, the Chinese Academy of Sciences. Polypropylene master-batch (Shanghai Eaststone New Material Development Co., Ltd) and MWCNTs (Shenzhen Nanotech Port Co., Ltd.) were used as received.

### Fabrication of LN/PP/MWCNT Films

The LN wafer polarizing process is as following: the bulky LN crystal is heated at 1423 to 1653 K, and a current density of 2–5 mA/mm^2^ and an electric field of 10 V/mm are applied simultaneously. The polarized LN crystal is cut into wafer or ball-milled into micro-particles with relatively uniform size about 1 μm.

PP masterbatch, 1 wt.% MWCNTs, and LN particles of different mass fractions (0, 1, 2, 3, 5, 8, 10 wt.%) were thoroughly mixed at room temperature. The mixture was then placed in a Dolylab OS Reactive Twin Screw Extruder System and then heated to 473 K and stirred for 5 min. The homogeneous mixture was placed in a laminator (XH-407) and heated to 473 K, and then the mixture is extruded and pressed between two metal splints under a pressure of 3 MPa for 5 minutes. After cooling to room temperature, a LN/PP/MWCNT composite film was successfully fabricated. The size and thickness of the film can be simply controlled with the accurate amount of input composite and pressure. Then, the copper wires are fastened to the tapes in advance to connect the pyroelectric composite sensors and measuring devices. Hot-pressing is a convenient and efficient method with the ability to produce tens of films at one time without size limitation.

### Characterization

The crystal phase structure of LN particles and conformation of the composite films are characterized by x-ray diffraction (XRD 7000, Shimadzu). The microscopic topography is characterized by a Dimension Icon system (Bruker, USA). The already fabricated LN/PP/MWCNT pyroelectric composite sensor is attached to the test area of the heating element and connected to an electrochemical workstation (CHI 660D, Shanghai Chenhua Instrument Co., Ltd.). A DC supplier (Keithley 2410 SourceMeter) is used to provide variable voltages to the heater chips, so that the composite film sensor closely adhered to the heater chips could work under different temperatures. The real-time current signals under different temperatures are monitored by using the I-T method of the electrochemical workstation analyzer.

## Results and Discussion

**Fig. 1:**
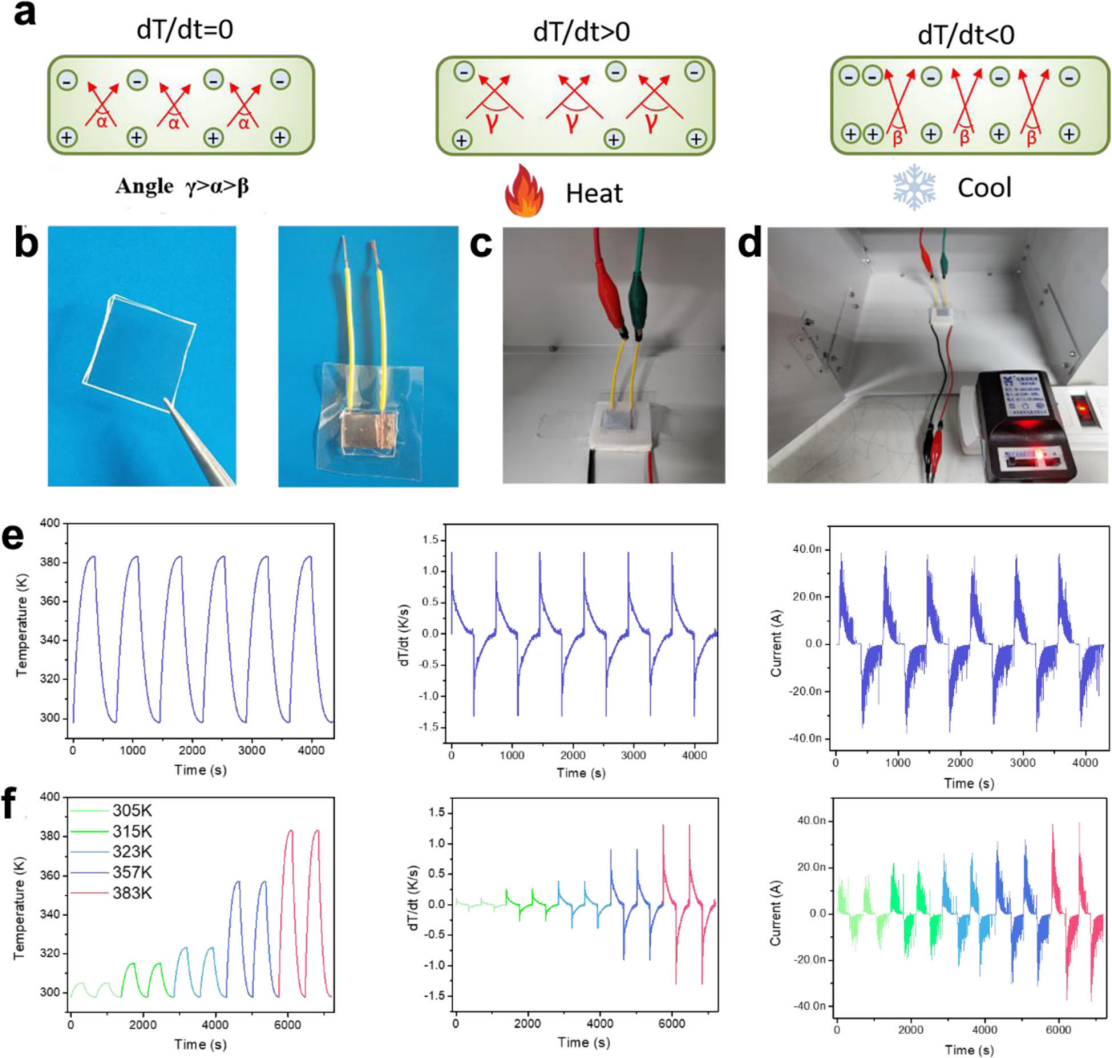
Pyroelectricity of LN crystal bulk***.*a** Schematic diagram of the pyroelectric working mechanism of the LN crystal wafer: the initial polarization state, the heated state, and the cooled state. Pyroelectric effects characterized using a heating element. **b** Photographs of the LN device with a piece of LN bulk wafer (2 cm × 2 cm). **c** A heating element used for the pyroelectric effect characterization. **d** Photographs of the heating element working with a DC power supply. **e** Pyroelectric current of the LN bulk under different temperatures. **f** Pyroelectric current of LN wafer with different change ranges and ramping rates of temperatures

Pyroelectric materials can exhibit spontaneous electric polarization, leading to the changes of the positive and negative charges at both sides of the crystals’ surface with the temperature changes. Below the Curie temperature, the spontaneous polarization of LN wafer or particles can be changed by heating or cooling, and electrostatic charges will be generated at both sides of the crystals as the schematic diagram shown in Fig. 1a. The generated charges can be harvested and converted into electrical current through a pre-designed circuit. The LN crystal wafer device (as shown in Fig. 1b–d) is attached on a heat plate, where the temperature of the heat plate can be controlled precisely. Figure 1e shows the cyclic changes in temperature of the LN device and the corresponding heating rate (dT/dt). According to Fig. 1e, a sharp pyroelectric current of ~ 40 nA is observed when the temperature increases from 298 to 383 K. When the temperature reversely decreases from 383 to 298 K, the obtained opposite current signals indicate that the measured currents are generated by the fabricated LN crystal wafer. Usually, the pyroelectric current *I* can be described as:

$$ I=\mathrm{pA}\left( dT/ dt\right) $$

where p is the pyroelectric coefficient of the material, A is the electrode area, and (dT/dt) is the ramping rate of the temperature.

We further set different changing ranges and ramping rates of the temperature, and the corresponding current signals change simultaneously, which are shown in Fig. 1f. It is obvious that the output currents of LN crystal wafer device will increase with an increasing changing range and ramping rate of the temperature. These results indicate that all the obtained signals shown in Fig. 1e are due to the pyroelectric effect of LN crystals, converting the pyroelectric charges into electrical current.

**Fig. 2:**
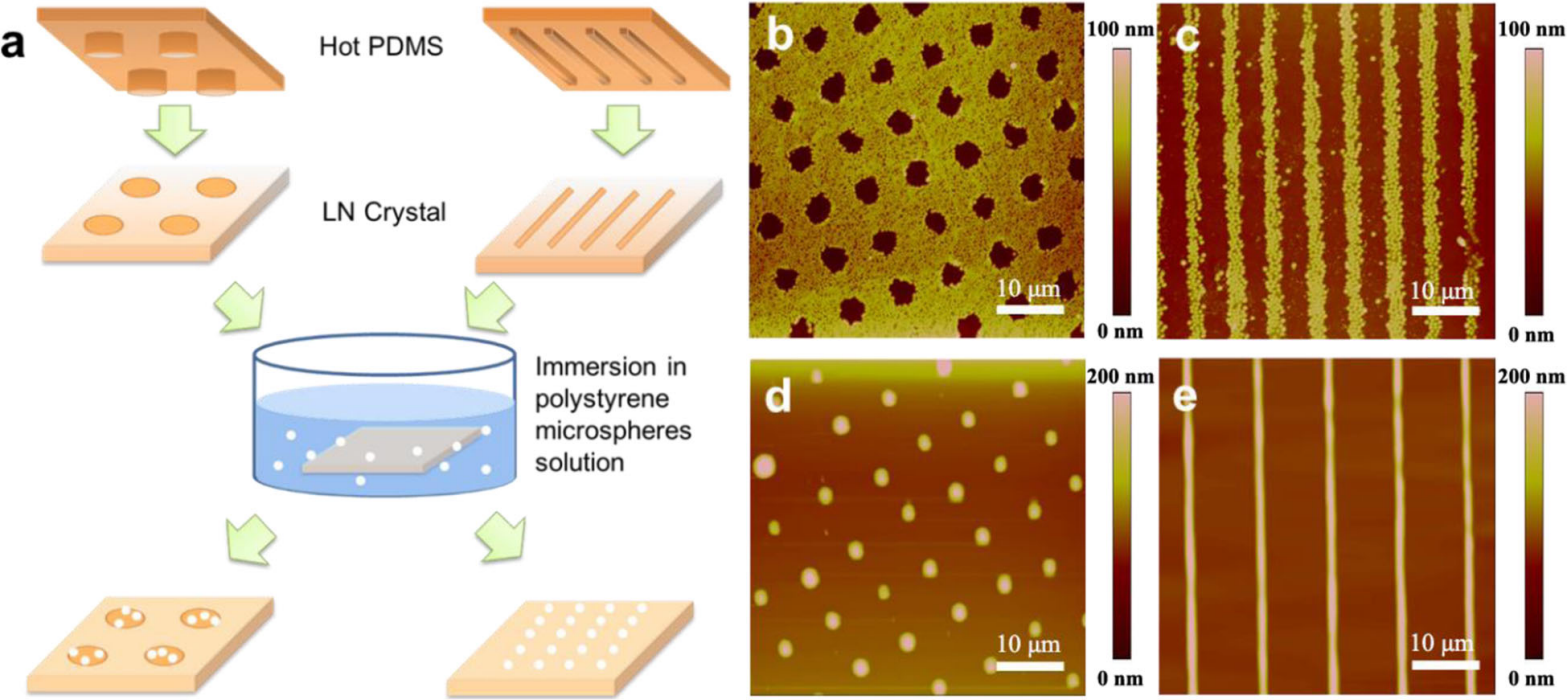
The electrostatic interaction driven self-assembly ability of particles or thin polymer films by pyroelectric effects in micro-scale. **a** Schematic illustration shows the procedure of PS nanoparticles and film patterning self-assembly by using pyroelectrostatic charge interaction on the heated LN crystal wafers. Sixty nanometers of PS nanoparticles are chosen to characterize the charge pattern. The patterned assembly of PS nanoparticles **b**, **c** and the electrohydrodynamic assembly of thin PS film **d**, **e** on the pyroelectri c charge areas featured by AFM

In order to exhibit the outstanding pyroelectric effects of LN crystal wafer, we further vividly used the electrostatic interaction driven self-assembly of particles or thin polymer films. The particles or thin polymer films could be patterned by the electrostatic interaction produced by the instantaneous pyroelectric charges. The schematic diagrams in Fig. 2a show the patterning process of pyroelectric charges on the surface of LN wafer and the electrostatic-induced self-assembly of PS micro-particles and thin film. A soft PDMS stamp is fabricated by using a contact printing method, in which the patterns are transferred to the PDMS from a patterned silicon wafer. When a hot PDMS stamp is contacted with LN wafer substrate, the heat transferred from PDMS stamp to the LN wafer, inducing a patterned micro-scale assembly of particles or thin polymer films on the charged area. Standard PS nanoparticles in organic solvent with 60 nm diameter and PS thin film (*M*_w_ = 5000) are chosen to form the patterns in self-assembly process. After taking the PS particles from the organic solvent (Fig. 2b, c) or spin-coating a thin layer (with a thickness of 100 nm) of PS film (Fig. 2d, e) onto the LN wafer, the electrostatic stress accumulated from the patterned pyroelectric surface charges drives the assembly of the particles and thin polymer film into microarrays at the charged area. Based on different charge patterns, which are fabricated by using different patterned PDMS stamps, we could observe various self-assembly structures. Circular periodic lattice is shown in Fig. 2b (or the complementary pattern in Fig. 2d), and the periodic linear stripes are shown in Fig. 2c, e.

Although the polarized LN bulk has outstanding pyroelectric effects, the fragility, inflexibility, and difficulty in processing will limit the application of its pyroelectric ability. We further fabricated a particle-polymer composite sensor, composed of LN crystal micro-particles and polypropylene (PP) matrix by hot-pressing procedures. The composite film could incorporate the excellent mechanical properties of PP matrix and the superior pyroelectric effects of LN particles. In order to obtain obvious current signals and reduce measuring errors caused by the electric resistance, a 1 wt.% concentration of MWCNTs are adopted and uniformly dispersed in the LN/PP composites by trial and error. Compared with LN/PP films, LN/PP/MWCNT pyroelectric composite film (PCF) flexible sensor has higher response signal, as shown in Fig. S1 of supporting information.

**Fig. 3:**
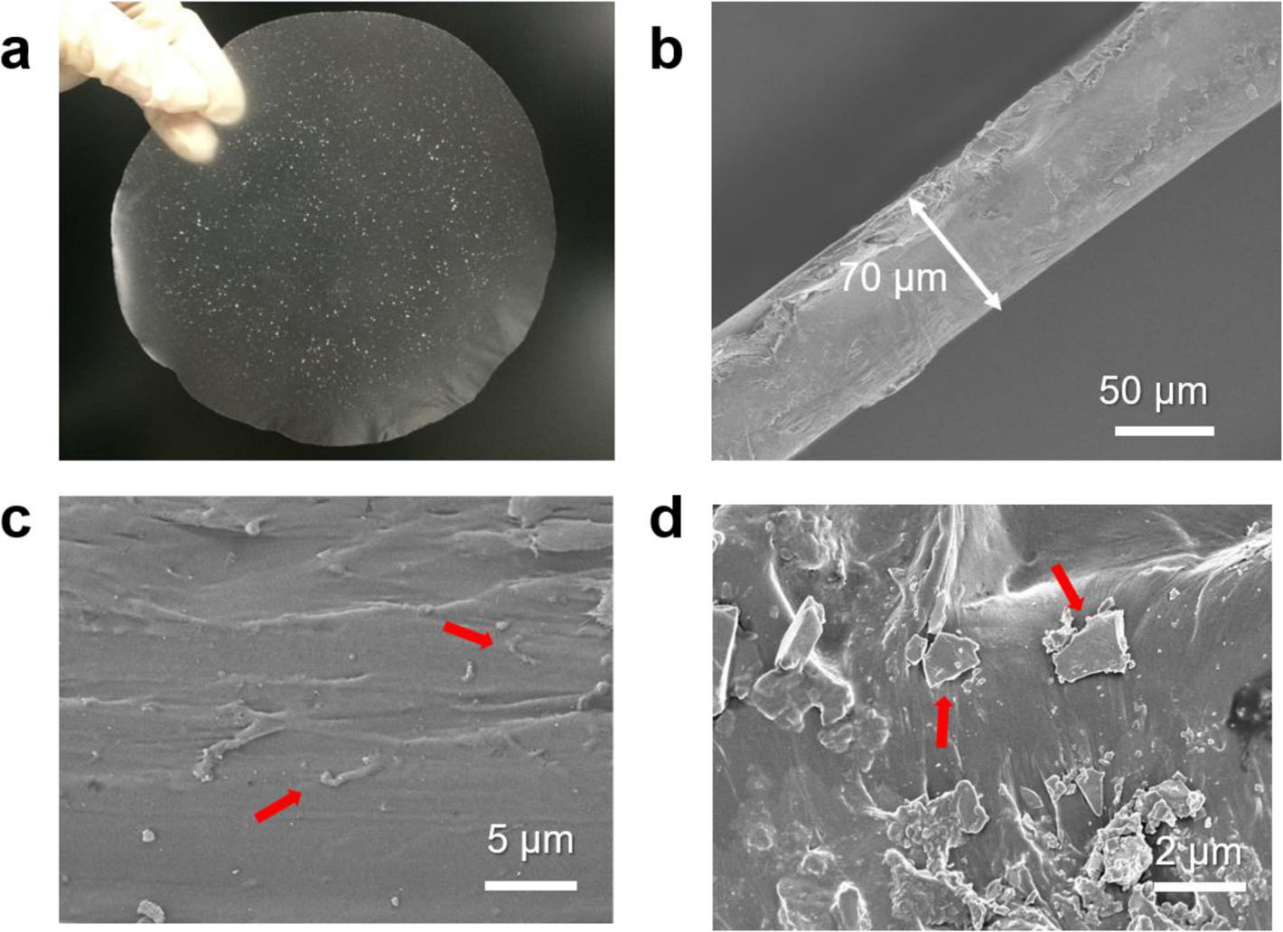
Photographs of the LN/PP/MWCNT film. **a** An intact piece of the LN/PP/MWCNT film. **b** SEM images of a cross section of the LN/PP/MWCNT film. The magnified cross section of where MWCNT **c** and LN particles **d** are indicated by red arrows

The SEM images of the fabricated LN/PP/MWCNT composite film are shown in Fig. 3. It could be observed that both LN micro-particle and MWCNT are uniformly dispersed in the composite films. The thickness of the LN/PP/MWCNT composite film is about 70 μm (as shown in Fig. 3b). The crystal phase structure of LN particles and conformation of the composite films are characterized by x-ray diffraction, as shown in Fig. S2 of supporting information.

**Fig. 4:**
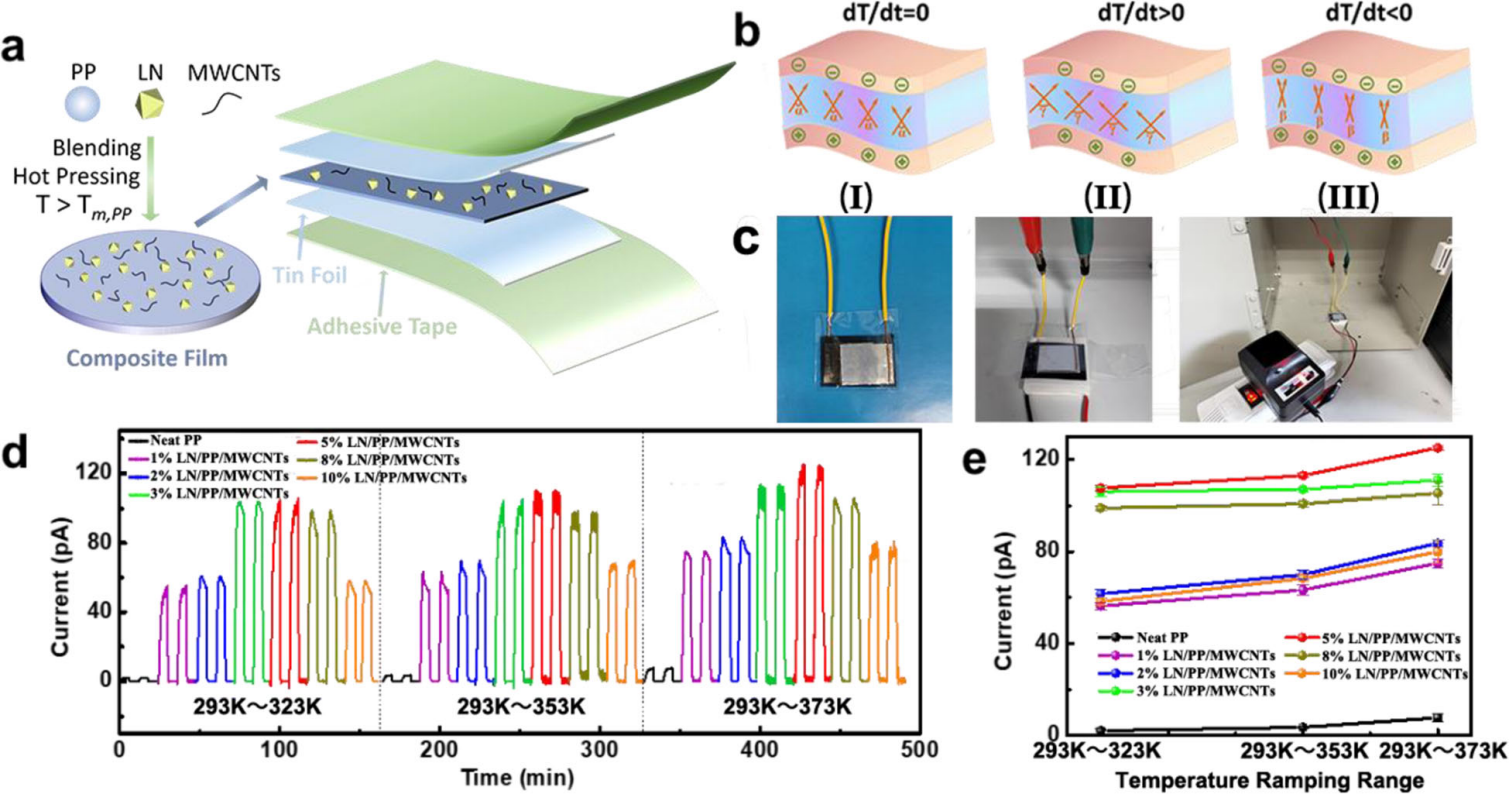
Pyroelectric effects of LN/PP/MWCNT nanocomposite. **a** Schematic diagram of the LN/PP/MWCNT composited film fabrication process. **b** Schematic illustration of the LN/PP/MWCNT pyroelectric nanogenerator structure and working mechanism: (I) the initial polarization state, (II) the heated, and (III) the cooled state of the LN/PP/MWCNT PCF. **c** Photographs of the heating element working with a DC power supply. **d**, **e** Pyroelectric current and trend of the LN/PP/MWCNT composited sensor with temperature ramping dependency and LN microparticles’ concentration dependency

The schematic fabrication process of the pyroelectric LN/PP/MWCNT film and sensor is shown in Fig. 4a; the heating-cooling procedure and the corresponding current changes are also schematically illustrated in Fig. 4b. The pyroelectric properties of the composited polymer are further investigated by monitoring the pyroelectric current signals of a LN/PP/MWCNT sensor. Pyroelectric currents with different LN concentration (0, 1, 2, 3, 5, 8, and 10 wt.%) and 1 wt.% MWCNTs are monitored by using an electrochemical station as shown in Fig. 4c, and the output currents are monitored and shown in Fig. 4d, e. Similar to LN crystal wafers, the PCF flexible sensor exhibits obvious temperature ramping dependence, which is shown in Fig. 4d. With the temperature ramping range continuously increasing from 293 ~323 K to 293 ~373 K, the output current increases obviously.

Moreover, the output current signals are closely related to the concentrations of LN micro-particles. According to Fig. 4e, the pyroelectric currents increase with the increasing of LN micro-particles’ concentration. When the temperature range from 293 to 373 K with the LN nanoparticles concentration of 5 wt.%, the largest pyroelectric current up to ~ 125 pA is observed. However, the pyroelectric effects begin to decrease once more than 5 wt.% LN particles are incorporated in the PP matrix. This phenomenon is probably due to the copolymer matrix disorganization caused by the excess LN nanoparticles. In addition, excess LN nanoparticles could also make the LN/PP/MWCNT composite film fragile and difficult to be hot-pressed. Therefore, it is recommended to choose the film containing 3 wt.% LN nanoparticles as an appropriate formula for further researches due to its better pyroelectric property, higher mechanical strength, and lower cost.

The polymer-based flexible films are successfully fabricated, and the pyroelectric properties are characterized quantitatively. The outstanding pyroelectric effects and the flexible property will make this composite feasibly to be used under many conditions such as sensors or energy harvesters since the shape of the films could be changed randomly. However, rigorous investigations should be conducted to study the mechanism and further applications of the pyroelectric effect.

## Conclusions

To sum up, we investigated the pyroelectric properties of LN crystal wafer and LN/PP/MWCNT composite. The polarized LN wafer shows outstanding pyroelectric effects under a moderate temperature, which could induce the self-assembly of PS micro-particles and thin films. We successfully fabricated a flexible LN/PP/MWCNT composite film with pyroelectric effects and outstanding mechanical properties. By monitoring the output currents under the stimulation of temperatures and the concentration of LN micro-particles, the pyroelectric effects are characterized, and the optimized concentration is recommended for subsequent researches. The perfect combination of pyroelectric properties of LN microparticles and the flexibility of the PP polymer will make it possible to be used as heat energy harvesters to supply electric energy and explore more applications.

## Supplementary information

**Additional file 1: Figure S1.** Comparison of neat PP, LN/PP and LN/PP/MWCNTs composited sensor pyroelectric current signals(1wt.% MWCNTs, 293K~353K).

**Additional file 2: Figure S2.** The crystal phase structure of lithium niobate particles and conformation of the composite films are characterized by X-ray diffraction.

## Data Availability

The datasets used or analyzed during the current study are available from the corresponding author on reasonable request.

## Abbreviations

LN: Lithium niobate
PP: Polypropylene
FEM: Ferroelectric materials
PDR: Pyroelectric detectors of radiation
PZT: Lead zirconate titanate piezoelectric ceramics
PVDF: Polyvinylidene fluoride
BaTiO_3_: Barium titanate
PDMS: Polydimethylsiloxane
PS: Polystyrene
AFM: Atomic force microscopy
PCF: Pyroelectric composite film
