# Supplementary material for: Studying the Pyroelectric Effects of LiNbO3 Modified Composites
Source: Nanoscale Res Lett. 2020 May 12;15:106. doi: 10.1186/s11671-020-03341-w (PMC7218041; doi:10.1186/s11671-020-03341-w)

**Fig. S1.** Comparison of neat PP, LN/PP and LN/PP/MWCNTs composited sensor pyroelectric current signals(1wt.% MWCNTs, 293K~353K)


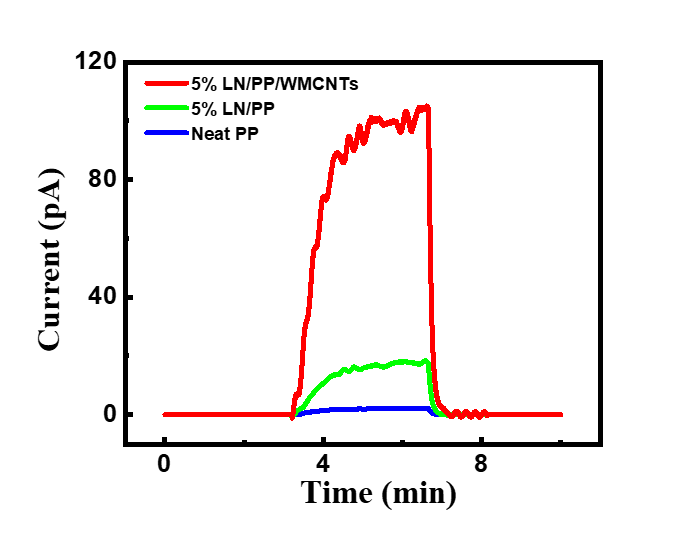

Supplement: Supplementary file 1 — Additional file 1: Figure S1. Comparison of neat PP, LN/PP and LN/PP/MWCNTs composited sensor pyroelectric current signals(1wt.% MWCNTs, 293K~353K). [file 11671_2020_3341_MOESM1_ESM.docx]
