# Supplementary material for: Studying the Pyroelectric Effects of LiNbO3 Modified Composites
Source: Nanoscale Res Lett. 2020 May 12;15:106. doi: 10.1186/s11671-020-03341-w (PMC7218041; doi:10.1186/s11671-020-03341-w)

**Fig. S2.** The crystal phase structure of lithium niobate particles and conformation of the composite films are characterized by X-ray diffraction
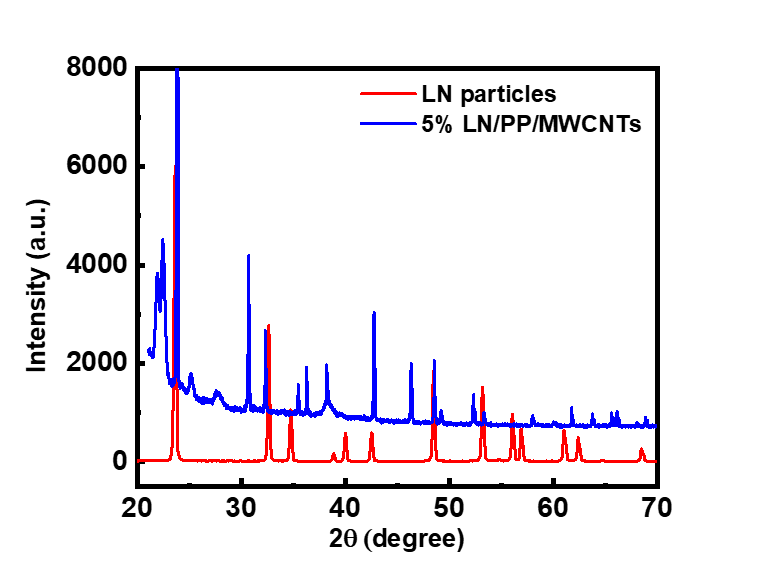

Supplement: Supplementary file 2 — Additional file 2: Figure S2. The crystal phase structure of lithium niobate particles and conformation of the composite films are characterized by X-ray diffraction. [file 11671_2020_3341_MOESM2_ESM.docx]
